# Is the relationship between problematic mobile phone use and mental health problems mediated by fear of missing out and escapism?

**DOI:** 10.1016/j.abrep.2021.100384

**Published:** 2021-10-08

**Authors:** Nevra Atış Akyol, Derya Atalan Ergin, Anna K. Krettmann, Cecilia A. Essau

**Affiliations:** a Sivas Cumhuriyet University, Turkey; b Ministry of Education, Turkey; c Centre for Applied Research and Assessment in Child and Adolescent Wellbeing, London, UK; d University of Roehampton, London, UK

**Keywords:** Problematic mobile phone use, Fear of missing out, Mental health problems, Worry, Pre-service preschool teachers

## Abstract

**Introduction:**

Individuals with problematic mobile phone use often report mental health problems, however, little is known about factors which mediate this association. Therefore, the aim of this study was to examine the mediating role of fear of missing out (FoMO) and escapism in an attempt to explain the relationship between problematic mobile phone use and mental health problems.

**Method:**

A total of 235 undergraduate students in Turkey participated in this study. These participants completed a set of questionnaires to measure fear of missing out, problematic mobile phone use, escapism, depression, anxiety, stress, and worry severity.

**Results:**

Participants with a high level of problematic mobile phone use also had a high level of mental health problems, FoMO, and escapism. The association between problematic mobile phone use and mental health problems was not mediated by FoMO and escapism.

**Conclusion:**

Personal characteristics such as emotional regulation and self-regulation need to be examined in future studies in order to determine their potential mediating role in explaining the association between problematic mobile phone use and mental health problems.

## Highlights

• Higher level of problematic mobile phone use was associated with higher level of mental health problems, Fear of Missing Out (FoMO) and escapism.
• Higher level of mental health problems was associated with higher level of FoMO.
• Higher level of FoMO and worry was associated with higher level of escapism.
• The association between problematic mobile phone use and mental health problems was not mediated by FoMO and escapism.

## 1 Introduction

Mobile phones have become an important part of our everyday lives. These digital devices have significantly changed the way we live, ranging from our social interaction to shopping opportunities. Particularly during the current pandemic, when people have to comply with preventative measures to contain the spread of COVID-19 (e.g., social distancing, lockdown), mobile phones or smartphones (these two terms are used interchangeably in this article) have played a key role in keeping family and friends connected. It is therefore not surprising that the rates of mobile phone ownership have grown exponentially in recent years. Globally, there are 5.27 billion mobile phone users in the world, which accounts for approximately 67.1% of the world’s population (Digital, 2021).

In Turkey, where this study took place, the number of mobile phone users are estimated to be 52.8 million users; this number is expected to increase to 56.4 million in 2023 (Statista, 2021). Cultural characteristics may be one of the determinants of individuals' mobile phone usage (Gao et al., 2020). In collectivist cultures where interpersonal relationships are encouraged, people use mobile phones more to engage in interpersonal interactions (Shin, 2014). People in individualistic cultures have different mobile phone usage patterns than people in collectivistic cultures (Panova et al., 2020). As reported by Lopez-Fernandez et al. (2017), individuals from collectivist cultures in Europe (including Spain and Italy) used smartphones to foster and maintain interpersonal communication; conversely, individuals from individualistic cultures (including the United Kingdom and Finland) used smartphones for professional/academic or leisure purposes. Turkey is a country that has collectivist cultural features (Newman et al., 2015), where feeling of interconnectedness is of importance; smartphones therefore play an important role in fostering and maintaining feeling of interconnectness with friends and family members.

Furthermore, the high number of mobile phone users in Turkey are most likely related to Turkey’s young population, with about 40% of its population being younger than 25 years (The World Factbook, 2021). According to Torlak, Spillan and Harcar (2011), young people made up the fastest-growing market for mobile phone in Turkey. Some of the reasons for this were related to more two-income families with fewer children, higher divorce rate where separated parents feeling emotionally obligated to give their children more material possessions, and the need of young people to strive towards “fitting in” or “being cool” (Torlak et al., 2011).

Although the use of smartphones offers a wide range of benefits, their overuse can be detrimental to physical health, psychological wellbeing, academic performance, and social relationship. Physically, overuse of mobile phone or problematic mobile phone use has been linked to neck, hand and shoulder pain (Haug et al., 2015; Elhai et al., 2017), headache (Korpinen & Pääkkönen, 2009), concentration difficulties (Thomee et al., 2011), physical inactivity (Barkley and Lepp, 2016, Fennell et al., 2019), and sleep disturbances (Thomee et al., 2011). Socially and academically, problematic mobile phone use have been linked to worsening of academic performance (Samaha & Hawi, 2016) and personal relationship (Seo et al., 2016), and well as to an increase in procrastination (Rozgonjuk et al., 2018). Psychologically, problematic mobile phone use has been linked to anxiety, depression (Elhai et al., 2017), and stress (Cao et al., 2018) for which the term ‘technostress’ or ‘techno-exhaustion’ has been used to describe smartphones users who become stressed, overwhelmed, and exhausted because to mobile phone use. Worry, as one of the key symptom of anxiety disorders and other disorders (e.g., depression) has also been linked with problematic mobile phone use.

Worry might be seem as one of the main feeling related to mental health problems (Anniko et al., 2019). People having high level of worry might be more prone to smart phone use (Elhai et al., 2019); however, studies that examined this relationship is lacking. Ruminations which is related to both smartphone use and worry, have also been found to be related to smartphone problem use (Elhai et al., 2018). In addition, nomophobia which is defined as fear of being detached from mobile phone connectivity, is associated with worry (Polat et al., 2021).

Given the high use of mobile phone, numerous studies have examined factors that lead to or are associated with problematic mobile phone use. One of the factors that have attracted research attention is fear of missing out (FoMO) which refers to the pervasive apprehension of missing out when one is absent and the need to continually know what other people are doing (Przybylski et al., 2013). This apprehensive feeling often triggers social surveillance behaviors such as tracking myriad status updates, photos, and videos in social media (Buglass et al., 2017). Since the first publication of the FoMO article by Przybylski et al. (2013), accumulative studies have reported FoMO to be positively associated with anxiety (Blackwell et al., 2017, Elhai et al., 2016) and depression (Elhai et al., 2016), whileas others have shown FoMO to predict of social media addiction (Blackwell et al., 2017). While informative, the studies above were mostly conducted among adults. The extent to which these findings could be replicated among young adults in a non-Western country (i.e, Turkey) is unclear. The present study focused on young adults (i.e., undergraduate students studying pre-service preschool teaching) as they have been identified as the most at risk for developing FOMO (JWT Intelligence, 2011). Furthermore, pre-service teachers have been reported to consider mobile phone as their basic survival needs such as organs, lovers, food, etc (Gezgin et al., 2019). As preschool teachers spend many hours with children and are responsible for educating them, it is important for teachers to be a role model for the children such as in promoting children's development (Denham et al., 2012, Lillvist et al., 2014, Pianta et al., 1997). Teacher’s expertise not only determine the quality of education they provide, but also contribute to children's development.

Given the high prevalence of problematic mobile phone use among young adults (Tao et al., 2020), it is important to determine the extent to which this is related to mental health problems so that appropriate prevention effort could be delivered to strengthen the mental health of students taking pre-service preschool degree/courses before they enter the teaching profession. Escapism has also been identified as one of the most important factors that has been associated with problematic mobile phone use and internet addiction (Panova and Alejandro, 2016, Hagström and Kaldo, 2014). The motive for escapism is to escape from stress and to avoid confronting stressful situations. Ohno (2016), in a study of Japanese found escapism to mediate psychological distress and internet addiction. In that study, ‘‘escape to peace of mind by empathy’’ was an important theme which was expressed by the adolescents as ‘‘I feel reassured by finding the same opinions and people who agree with me in the Social media,’’ and ‘‘I can get so many comments that it is calming.’’. The author went on to describe that when it is difficult to cope with stressors which require empathy in real life, then social media may help to escape from challenges that cannot be understood or empathized with other people.

The present study will contribute to knowledge on factors related to problematic mobile phone use among young people in a non-Western country. The specific aim was to investigate the relationship between problematic mobile phone use, mental health problems (depression, anxiety, stress, and worry), and dysfunctional Internet usage (FoMO and escapism) among Turkish university students. Another aim was to explore the mediating role of FoMO and escapism in explaining the association between problematic mobile phone use and mental health problems.

**Fig. 1:**
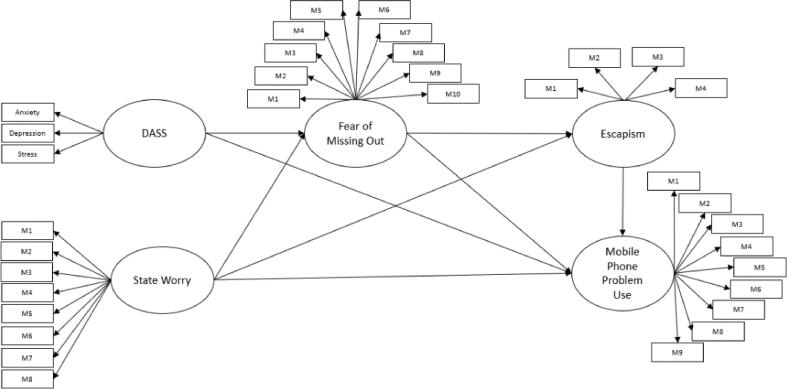
Hypothesis model between DASS, state worry, FoMO, Escapism and mobile phone problem use. *Note:* DASS = Depression, Anxiety and Stress Scale; FoMO = Fear of Missing Out.

Based on findings of previous studies described above, the specific hypotheses to be tested were: (1) Mental health problems (depression, anxiety, stress, and worry) was associated with problematic mobile phone use. (2) FoMO mediated the association between mental health problems (depression, anxiety, stress, and worry) and problematic mobile phone use. (3) Escapism mediated the association between FoMO and problematic mobile phone use. (4) Mental health problems (depression, anxiety, stress, and worry) was directly associated with problematic mobile phone use through multiple mediating roles of FoMO and escapism. The guiding model that illustrates the link between mental health problems (i.e., depression, anxiety, stress), worry, FoMO, escapism and problematic mobile phone use is shown in Fig. 1.

## 2 Material and methods

### 2.1 Participants

A total of 235 undergraduate pre-service preschool teachers participated in this study; they range in age from 18 to 25 years (M = 21.72, SD = 2.73). Most students were females (84.7%) and were unmarried (95.8%). The participants were recruited from the department of preschool teacher education in six universities in Turkey. Students were recruited through convenience sampling.

### 2.2 Procedure

The study was approved by the Ethic Committee at KTO Karatay University, Turkey and was conducted in accordance with the 1975 Declaration of Helsinki. The study was conducted through a web-based online survey using Google Forms. Participants first completed a consent form in which they agreed to participate in the study and stated that they were over 18 years. The consent form clearly stated the voluntary nature to this study. They were able to stop participating in the study or withdraw the data by closing their browser. The participants were informed that their participation was completely anonymous as no personally identifying information was collected. The link to the online survey were sent to the departments responsible for preschool teacher training in the six universities in Turkey for their relevant staff members to distribute to their students.

### 2.3 Measures

Fear of Missing Out Scale (FoMOS; Przybylski et al., 2013; Turkish version: Gökler et al. (2016) was used to measure fear of missing out. Participants respond to statements measuring the extent to which they fear missing out on various events or experiences. The FoMOS consists of 10 items and participants were asked to indicate the extent to which they fear missing out on various events or experiences. Each item can be rated on a five-point Likert type ranging from ''not at all true of me (1)'' to '' extremely true of me (5)''. A higher score indicated a higher level of FoMOS. The Cronbach's Alpha in the present study was 0.80.

The 10-item Mobile Phone Problem Use Scale (MPPUS-10; Foerster et al. (2015) was used to assess problematic mobile phone use, including symptoms of craving and withdrawal. It contains 10 items which can be rated on a 10-point Likert scale ranging from 1 (“not true at all”) to 10 (“extremely true”). The total scores could be obtained by adding all the items, with higher score indicating a higher level of mobile phone problematic usage. In the present study, one item (item five) has a lower factor loading, therefore it was removed from the analysis. The Cronbach's Alpha found in the present study was 0.84.

Escapism Scale (Gao et al.2017) was used to measure unpleasant realities, problems, and pressures using internet services or apps. The scale consists of four items which are to be rated on a four-point Likert scale ranging from “Strongly disagree (1)” to “Strongly agree (4)”. A higher score indicates a higher level of escapism. Cronbach's Alpha found in the present study was 0.89.

Depression and Anxiety Stress Scale-21 (DASS-21; Lavibond & Lavibond, 1995; Turkish version; Yılmaz et al., 2017) was used to measure depression, anxiety, and stress. Participants indicates how much each of the 21 items apply to them over the past week over four-point Likert Scale, ranging from 0 “did not apply to me at all” to 3 “applied to me very much or most of the time”. A total score will be calculated by summing all the 21 items. The Cronbach's Alpha in the present study was 0.94.

The Penn State Worry Questionnaire—Abbreviated (PSWQ-A; Hopko et al., 2003) was used to measure worry severity. It contains 8 items which can be rated on a five-point Likert scale ranging from “not at all typical of me (1)” to “very typical of me (5).” A higher score indicated a higher level of worry. The Cronbach's Alpha in the present study was 0.94.

### 2.4 Statistical analyses

Prior to the planned analyses, the data were checked for normality, outliers, and missing values. All assumptions were met for further analysis. Pearson correlation coefficients were calculated to examine the relationships between all the study variables. Subsequently, structural equation modelling (SEM) was conducted. Independent variables in the model (Fig. 1) were direct and indirect involvement in mental health problems (depression, anxiety, stress and worry); FoMO and escapism were two serial mediators, and mobile phone problem use was the dependent variables.

## 3 Results

Descriptive statistics are presented for the study variables in Table 1, including their means and standard deviations. Table 1 also presents bivariate correlations between the study variables. Problematic mobile phone use was significantly correlated with FoMO, escapism, and all the three subscales of DASS-21 (i.e., depression, anxiety and stress). Significant positive correlations were also found between all the other study variables.

**Table 1:** Descriptive statistics and the correlation coefficients for the study variables.

	1	2	3	4	5	6	7	M	SD
1. Fear of Missing Out	1							27.77	7.05
2. Problematic Mobile Phone Use	0.45^***^	1						41.36	16.65
3. Escapism	0.33^***^	0.52^***^	1					8.65	3.79
4. DASS-21 (total scores)	0.36^***^	0.49^***^	0.38^***^	1				25.71	14.91
5. Anxiety (DASS-21)	0.35*^**^	0.42^***^	0.30^***^	0.89^***^	1			6.47	5.29
6. Depression (DASS-21)	0.32^***^	0.45^***^	0.39^***^	0.90^***^	0.70^***^	1		8.39	5.62
7. Stress (DASS-21)	0.31^***^	0.45^***^	0.34^***^	0.91^***^	0.72^***^	0.74^***^	1	10.85	5.60

*p < .05, **p < .01, ***p < .001; DASS-21 = Depression, Anxiety and Stress Scale

To examine the pathways that may link involvement in depression, anxiety, stress and worry and problematic mobile phone use via FoMO and escapism, and serially via FoMO and escapism were tested for DASS-21 (depression, anxiety, stress) and worry.

Table 2 shows the total and direct effects on FoMO, escapism and mobile phone problem use. The results of the causal relationships model showed a good overall fit: χ2 = 1075.839, df = 513, p ≤ 0.001; RMSEA = 0.06 [IC = 0.068–0.074]; CFI = 0.88; TLI = 0.87 (Table 2). When FoMO was regressed on DASS-21 and worry, 17.7% of variance in FoMO can be explained. Higher level of DASS-21 (β = 0.21**, *p* < .05) and worry (β = 0.36**, *p* < .05) were associated with higher level of FoMO. In total effect model for escapism, DASS-21, worry and FoMO, the model accounted for 29.9% of variance in escapism. Higher level of FoMO (β = 0.21*, *p* < .05) and worry (β = 0.21*, *p* < .05) were associated with higher level of escapism. However, no relationship was found between escapism and DASS-21 (β = 0.09, *p* > .05).

**Table 2:** Standardized coefficients for total and direct effects on FoMO, escapism and problematic mobile phone use in the serial mediation model.

Variables	FoMO	Escapism		problematic mobile phone use	
	β	β total	β direct	β total	β direct
DASS-21	0.23*	0.14	0.09	0.13*	0.12***
Worry	0.21*	0.47***	0.36**	0.03	0.03
FoMO			0.21**	0.17**	0.12*
Escapism					0.20***
R^2^	0.17	0.29		0.13	

*p < .05, **p < .01, ***p < .001. DASS-21 = Depression, Anxiety and Stress Scale; FoMO = Fear of Missing Out

Table 3 shows the indirect effect on mobile phone problem use via pathways and the 95% confidence intervals (CIs) for mental health problems. Total effect model for problematic mobile phone use accounted for 13.5% variance in problematic mobile phone use (Table 3). Higher level of DASS-21 (β = 0.12***, p < .001), FoMO (β = 0.12*, p < .05) and escapism (β = 0.20***, *p* < .001) were associated with higher level of problematic mobile phone use. However, there was no direct effect of worry (β = 0.03, *p* > .05) on problematic mobile phone use.

**Table 3:** Total, individual, and serial indirect effects for DASS-21 and worry on problematic mobile phone use.

Variables	Mediators	Indirect	95%-confidence interval	
			LL	UL
DASS-21	FoMO	0.03	−0.002	0.022
	FoMO & Escapism	0.01	0.000	0.007
	Escapism	0.02	−0.006	0.018
Worry	FoMO	0.07	−0.009	0.080
	FoMO & Escapism	0.01	−0.003	0.27
	Escapism	0.07	0.024	0.164

*p < .05, **p < .01, ***p < .001. FoMO = Fear of Missing Out; DASS-21 = Depression, Anxiety and Stress Scale

**Fig. 2:**
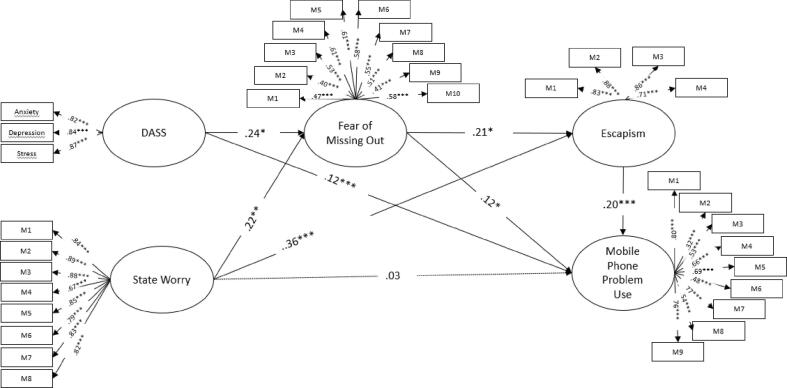
Structural Model between DASS, state worry, FoMO, Escapism and mobile phone problem use. *Note:* DASS = Depression, Anxiety and Stress Scale; FoMO = Fear of Missing Out.

Fig. 2 shows the standardized path coefficient for the serial mediation model. Our mediation analysis results (Fig. 2) showed no significant indirect effects on problematic mobile phone use via FoMO and escapism (Indirect effect_FoMO_ = 0.03, CI = -0.00-0.02; Indirect effect_Escapism_ = 0.02, CI = 0.00-0.02) for DASS-21 (Indirect effect_FoMO_ = 0.03, CI = -0.00-0.02; Indirect effect_Escapism_ = 0.02, CI = 0.00-0.02) and worry (Indirect effect_FoMO_ = 0.07, CI = -0.01-0.08; Indirect effect_Escapism_ = 0.07, CI = 0.02-0.16). Indirect effects on problematic mobile phone use via FoMO and escapism were not significant for DASS-21 (Indirect effect = 0.01, CI = 0.00-0.01) and worry (Indirect effect = 0.01, CI = 0.02-0.16).

## 4 Discussion

Our study examined the relationship between problematic mobile phone use, mental health problems, FoMO and escapism among Turkish university students, and explored the mediating role of FoMO and escapism in explaining these associations. Our results highlighted the direct effect of mental health problems, FoMO, and escapism on mobile phone use. These findings supported previous research which has shown a direct relationship between FoMO (Fuster et al., 2017), mental health problems (Thomée, 2018), and mobile phone use. Additionally, higher levels of FoMO and state worry were found to be associated with higher levels of escapism. However, there was no significant association between mental health problems and escapism. Previous research has shown that there is a link between escapism and mental health problems (Fernandes et al., 2020, Ohno, 2016). The reasons for this inconsistent finding were unclear, perhaps they might be related to the population being examined. Participants from the study by Fernandes et al. (2020) were young people in India, Malaysia, Mexico and the UK, whereas participants from Ohno’s study (2016) were senior high school students in Japan. Speculatively, cultural values and other environmental factors could have some influence in the link between between escapism and mental health problems. Future studies are needed to explore this speculation further.

Structural models provide the opportunity to examine the relationships between all variables in a model (Kline, 2011). In the present study, the relationship between depression, anxiety and stress as measured using the DASS-21 and escapism was not supported in the model, although it is directly significant. This result may emphasised the importance of other variables and their relationships. Although there are some studies showing the association between internet addiction and escapism or playing online games (Billieux et al., 2011), the current study is the first to examine the association between FoMO, state worry, mobile phone use, and escapism. Unlike previous studies (Elhai et al., 2019), state worry was not related to mobile phone problem use. Reason for this inconsistent finding was unclear although it could be related to the unique circumstances that young people are experiencing during Covid-19 pandemic. The worry of individuals increased in during the pandemic (Essau and de la Torre-Luque, 2021, Pan et al., 2021) and subject of worries was related to COVID-19 (Taylor et al., 2020). People tried to get social support and be connected to their families and friends using smartphones; at the same time people obtain information about the COVID-19 by searching the internet by means of their smartphones. Research emphasizes that the purpose of Internet use is related to mental health (Wallinheimo & Evans, 2021). It would also be conceivable that smartphone use for different purposes have a link with level of worry. Consequently, the purpose of use could play a crucial role between worry and mobile phone problem use. Our finding also showed higher levels of mental health problems and state worry to be associated with higher levels of FoMO. These findings were consistent with the results of previous research (Baiano et al., 2020, Hunt et al., 2018).

In line with our hypothesis and in agreement with previous studies (Cao et al., 2018, Elhai et al., 2017), the results showed that the participants who had high level of mental health problems, FoMO and escapism also had a high level of problematic mobile phone use. The reason for this association is not clear. It could be that anxiety, depression or stress may lead to problematic mobile phone use as way to cope with stress (Elhai et al., 2017), or that problematic mobile phone use could cause these mental health problems (Demirci et al., 2015). However, since of the cross-sectional nature of our study design, it is not possible to establish any causality in this association.

Our finding on the association between FoMO and mental health problems also replicated findings of previous studies (Blackwell et al., 2017, Elhai et al., 2016). While is beyond the scope of this study to examine the reasons for association, several authors have speculated that as FoMO is related to fear of being socially excluded plays, this fear could produces a sense of loss of belongingness, which in turn could lead to anxiety (Abel et al., 2016). Future studies are needed to confirm this hypothesis using studies with longitudinal design.

Our hypothesis that FoMO and escapism would mediate the association between problematic mobile phone use and mental health problems were not supported. It is plausible that other mechanism might be involved such as emotion regulation. For example, a study by Elhai et al. (2016) has shown emotional suppression to have mediated the association between problematic smartphone use and anxiety. According to these authors (Elhai et al., 2016) emotional suppression may have disrupted adaptive processing of emotions, which in turn is associated with greater depression.

Although there is an extensive literature in both Turkey and other countries highlighting the relationship between mental health, FoMO, and escapism, there are also some researches that have found no relationship between these variables (Balta et al., 2020, Chen and Chang, 2019). An alternative explanation could be related to these findings. In particular, women are more likely to have mental health problems than men (Chen & Chang, 2019). They are also more prone to problematic smartphone use (Van Deursen et al., 2015). In the current study, the proportion of female participants was higher than that of males. The mediating effect, which was not showed in the study, might be due to the strong association between mental health problems and smartphone problem use in women.

Our study is not without methodological limitations. Firstly, a convenience sample of university students who were studying pre-school education were used which may affect the generalizability of our findings. Furthermore, most of the participants (85%) were females, which was a reflection of the profile of students who study pre-school education. Secondly, the data were collected using self-report questionnaires which might be subjected to social desirability. Third, all measurement tools developed in the western culture and adapted to Turkish. Future studies need to develope scales that are specifically use to measure mental health problems, FoMO and escapism in the context of the Turkish culture. Finally, the study used a cross-sectional research design which does not allow for testing causality.

In spite of these shortcomings, our findings provided support on the association between problematic mobile phone use, mental health problems, FoMO and escapism. Future studies are needed to explore further factors that mediate the association problematic mobile phone use and mental health problems. Some potential candidates include personal characteristics such as impulsivity, emotion regulation, and self-regulation.

To conclude, our findings that the participants who had high level of mental health problems, FoMO and escapism also had a high level of problematic mobile phone use have important implication for pre-service teacher training in Turkey. Especially, as we come out of the pandemic, efforts are needed to develop programmes that can be used to strengthen psychological resilience of students in pre-service teacher training.

## CRediT authorship contribution statement

All the authors substantially contributed to this manuscript. NAA: Data curation, Project administration, Investigation, Resources, Data analysis, Writing - review. DAE: Methodology, Software, Visualation, Formal analysis and interpreted the results, review. AKK: Conceptualization, Writing-original draft, Writing - review & editing. CAE: Supervision, Critique the output for important intellectual content, Provided theoretical support. NAA and AKK shared first authorship.

## Declaration of Competing Interest

The authors declare that they have no known competing financial interests or personal relationships that could have appeared to influence the work reported in this paper.
